# First Report of Two-Spot Cotton Leafhopper (*Amrasca biguttula* Ishida) (Hemiptera: Cicadellidae) on Commercial Cotton in the Southeastern United States

**DOI:** 10.3390/insects16090966

**Published:** 2025-09-15

**Authors:** Isaac L. Esquivel, Tim Bryant, Sean Malone, Alana L. Jacobson, Scott H. Graham, Paulo S. Gimenez-Cremonez, Phillip Roberts, Silvana Paula-Moreas, Dominic Reisig, Anders Huseth, Jeremy Greene, Francis P. F. Reay-Jones, Sally Taylor

**Affiliations:** 1 Department of Entomology & Nematology, North Florida Research and Education Center, Institute of Food and Agricultural Services, University of Florida, Quincy, FL 32351, USA; 2 Department of Entomology, Tidewater Agricultural Research and Extension Center, Virginia Tech, Suffolk, VA 23437, USA; 3 Department of Entomology and Plant Pathology, Auburn University, Auburn, AL 36849, USAcremonez@auburn.edu (P.S.G.-C.); 4 Department of Entomology, University of Georgia, CAES Campus, Tifton, GA 31793, USA; 5 Department of Entomology & Nematology, West Florida Research and Education Center, Institute of Food and Agricultural Services, University of Florida, Jay, FL 32565, USA; 6 Department of Entomology and Plant Pathology, North Carolina State University, Vernon G. James Research and Extension Center, Plymouth, NC 27962, USA; ddreisig@ncsu.edu; 7 Michigan State University, Department of Entomology, East Lansing, MI 48824, USA; huseth@msu.edu; 8 Department of Plant and Environmental Sciences, Edisto Research and Education Center, Clemson University, Blackville, SC 29817, USA; greene4@clemson.edu; 9 Department of Plant and Environmental Sciences, Pee Dee Research and Education Center, Clemson University, Florence, SC 29506, USA; 10 Cotton Incorporated, Cary, NC 27513, USA

**Keywords:** invasive species, *Gossypium hirsutum*, IPM, range expansion

## Abstract

The two-spot cotton leafhopper, *Amrasca biguttula* (Ishida, 1913) (Hemiptera: Cicadellidae), is a polyphagous pest native to the Indian subcontinent, where it is a significant pest of cotton (*Gossypium* spp.), okra (*Abelmoschus esculentus*, Moench), and other crops. At adult and immature stages, they feed on leaf cell contents, causing characteristic “hopperburn” symptoms and yield losses of up to 40% in cotton in its native range. In July 2025, *A. biguttula* was detected for the first time during the cotton growing season in commercial and experimental fields across multiple counties in Alabama, Florida, Georgia, and South Carolina. Identification was confirmed through morphological examination of diagnostic external features and male genitalia. Within four weeks, the pest was found in 101 counties, with injury symptoms ranging from mild chlorosis to severe necrosis and defoliation. Given the southeastern United States’ average of 979,339 hectares of cotton valued at USD 1.7–USD 2.8 billion annually, the potential for economic impact is considerable. Rapid symptom development, broad host range, and multiple generations per year underscore the need for coordinated monitoring and research to determine preliminary control measures and to identify potential alternative hosts. This report documents the first confirmed occurrence of *A. biguttula* in U.S. cotton and highlights its potential as an established recurring pest threat in the region.

## Simple Summary

The two-spot cotton leafhopper, an invasive pest, has been detected on cotton in multiple states within the southeastern United States during the 2025 growing season.

## 1. Introduction

The two-spot cotton leafhopper, *Amrasca biguttula* (Ishida, 1913) (Hemiptera: Cicadellidae), or cotton jassid, is a polyphagous pest of numerous economically important crops and horticultural plants. Its native range extends from Iran to Japan and South Asia to Indonesia, with records from Afghanistan, Vietnam, China, Taiwan, and Guam [1]. Both adults and nymphs feed on plant sap, primarily on the undersides of leaves, causing hopperburn symptoms such as chlorosis, necrosis, and leaf margin rolling. Feeding also results in honeydew deposition, fostering sooty mold growth that reduces photosynthesis [2]. Severe infestations stunt plant growth, cause defoliation, and lead to premature drop of buds, flowers, and fruitlets, ultimately reducing yield and quality [3].

In its native range, *A. biguttula* is a significant economic pest of several malvaceous crops, including cotton (*Gossypium* spp.) and okra (*Abelmoschus esculentus* Moench). The insect colonizes cotton at different stages of development, causing significant stunting/plant death in early cotton, resulting in 20–40% yield loss [4]. In okra and eggplant, *Solanum melongena* L., *A. biguttula* damages the plant from the seedling through to the fruiting stages, causing yield losses from 50 to 75% [5]. It also attacks potato (*Solanum tuberosum* L.), sunflowers (*Helianthus annuus* L.), hibiscus (*Hibiscus sabdariffa* Rottler), and legume crops [1]. Outside of its native range, *A. biguttula* is adventive in West Africa, where it has now been detected in multiple countries, with a significant impact on cotton production [5,6]. While the exact economic impacts of *A. biguttula* in its native range have not been quantified extensively, a recent outbreak in Africa indicated an economic loss of XOF 34 billion (USD 59 million) in Côte d’Ivoire and XOF 65 billion (USD 105 million) in Burkina Faso [7].

There had not been an occurrence of *A. biguttula* in the western hemisphere until 2023, when populations were detected on cotton and eggplant in Puerto Rico [8]. This detection immediately caused concern for cotton production in the southeastern United States, given its relative proximity. Alabama, Florida, Georgia, North Carolina, South Carolina, and Virginia averaged ~979,339 hectares of cotton annually from 2022 to 2024, with a projected ~728,434 hectares in 2025 [9]. The economic importance of cotton in this region heightens the potential impact of *A. biguttula* establishment, given its significant impact on cotton in its native range and documented yield losses elsewhere. In late fall of 2024, it was detected for the first time in Florida through the Florida Department of Agriculture and Consumer Services’ Cooperative Agricultural Pest Surveys’ monitoring network [10]. *Amrasca biguttula* was confirmed in 16 counties, primarily in southern Florida, with 1 county in the panhandle, and 33 positive sites had active populations. Specimens were collected from cotton, common wireweed, *Sida acuta* Burm. f., black nightshade, *Solanum nigrum* L., eggplant, hollyhock, *Alcea* spp., and *Hibiscus* spp. [11]. It was unknown whether *A. biguttula* would be able to overwinter and persist in this new range.

In July of 2025, populations of *A. biguttula* were detected on commercial and experimental cotton in multiple counties across the Florida panhandle. Following this initial detection in Florida, *A. biguttula* was detected in Alabama (AL), Georgia (GA), and South Carolina (SC). It was not detected in fields sampled in Virginia (VA) and North Carolina (NC). Here, we document the first confirmed records of *A. biguttula* during the cotton growing season across the southeastern United States and discuss its associated plant injury activity and potential establishment as a new and emerging pest for U.S. cotton.

## 2. Materials and Methods

### Survey and Collection of Amrasca biguttula

A regional monitoring effort was established in AL, FL, GA, NC, SC, and VA to monitor for this pest during the 2025 cotton growing season. Multiple locations were monitored using sweep nets and visual samples every 1–2 weeks after cotton was planted. On 3 July 2025, a University of Florida (UF) Extension Specialist was notified of a potential *A. biguttula* infestation in a commercial cotton field in Gilchrist County, Florida. Specimens were collected by hand, vacuum, and sweep net and placed in 95% ethanol (Decon Labs, King of Prussia, United States) for identification and verification. Following this initial detection, with collaboration from cotton entomologists from AL, FL, GA, NC, SC, and VA, additional commercial and experimental cotton fields were scouted for *A. biguttula*. All suspected *A. biguttula* specimens were preserved in 70% ethanol (Decon Labs, King of Prussia, United States) for positive identification.

The collected adult specimens were identified under a stereomicroscope at the UF Agronomic & Forage Crops Laboratory using the species-specific keys [12,13,14]. Abdomens from the male adult specimens were dissected from the insect, then cleared with 10% KOH (Sigma-Aldrich, Burlington, NJ, USA) and slide-mounted for visualization of the genitalia for species determination. Images of adult specimens and male genitalia were obtained with a Keyence VHX-X1 digital microscope imaging platform (Keyence, Osaka, Japan).

## 3. Results

### Survey, Collection, and Identification of Amrasca biguttula

**Figure 1:**
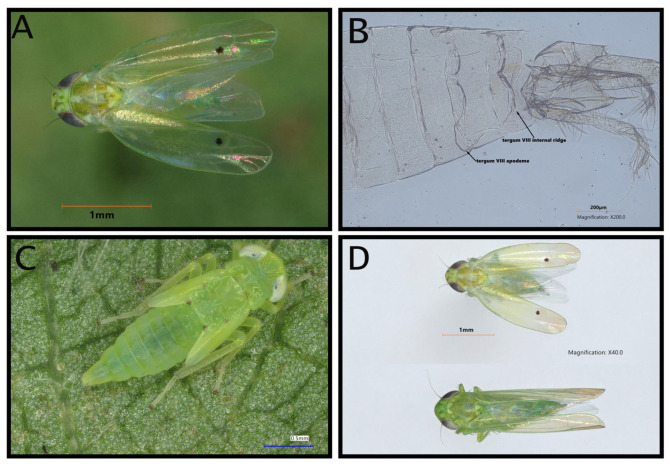
Adult *A. bigutulla* with two distinct black dots on the head and wing (**A**), dissected and cleared male abdomen indicating the pair of apodemes extending into tergum VII and tergum VIII with arched internal ridges (**B**), 5th instar nymph with markings on wing pads (**C**), and comparison of *A. bigutulla* and a similar looking adult potato leafhopper (**D**).

Specimens collected from commercial and experimental cotton fields were positively identified as *A. biguttula*. by Isaac L. Esquivel (University of Florida) and further verified by Susan Halbert (Florida Department of Agriculture and Consumer Services Division of Plant Industry). They were first identified based on the presence of two black spots on the apical cell of the forewings and two black spots on the crown of the head (Figure 1A). Examination of male genitalia further confirmed *A. biguttula* by a pair of lateral apodemes extending into segment VI and tergum VIII with a pair of arched internal ridges (Figure 1B) [15].

Initial detection in Florida commercial cotton was on 3 July 2025 by a crop consultant from Gilchrist Co., who sent photos of nymphs they had not seen in cotton before. While the potato leafhopper, *Empoasca fabae* (Harris), feeds on peanut and is sometimes found in cotton, nymphs are not often seen on cotton. On 7 and 8 July, *A. biguttula* were detected on experimental cotton plots at the UF North Florida Research and Education Center (Quincy, FL, USA) and on commercial cotton in Jackson and Calhoun Counties. First detection in South Carolina cotton was on 7 July in Barnwell County. In Georgia, it was first detected on Okra in Seminole County before it was found in cotton on 9 July. It was first detected in Alabama on 19 June from sweep net samples on cotton that had not been processed and confirmed until mid-July.

**Figure 2:**
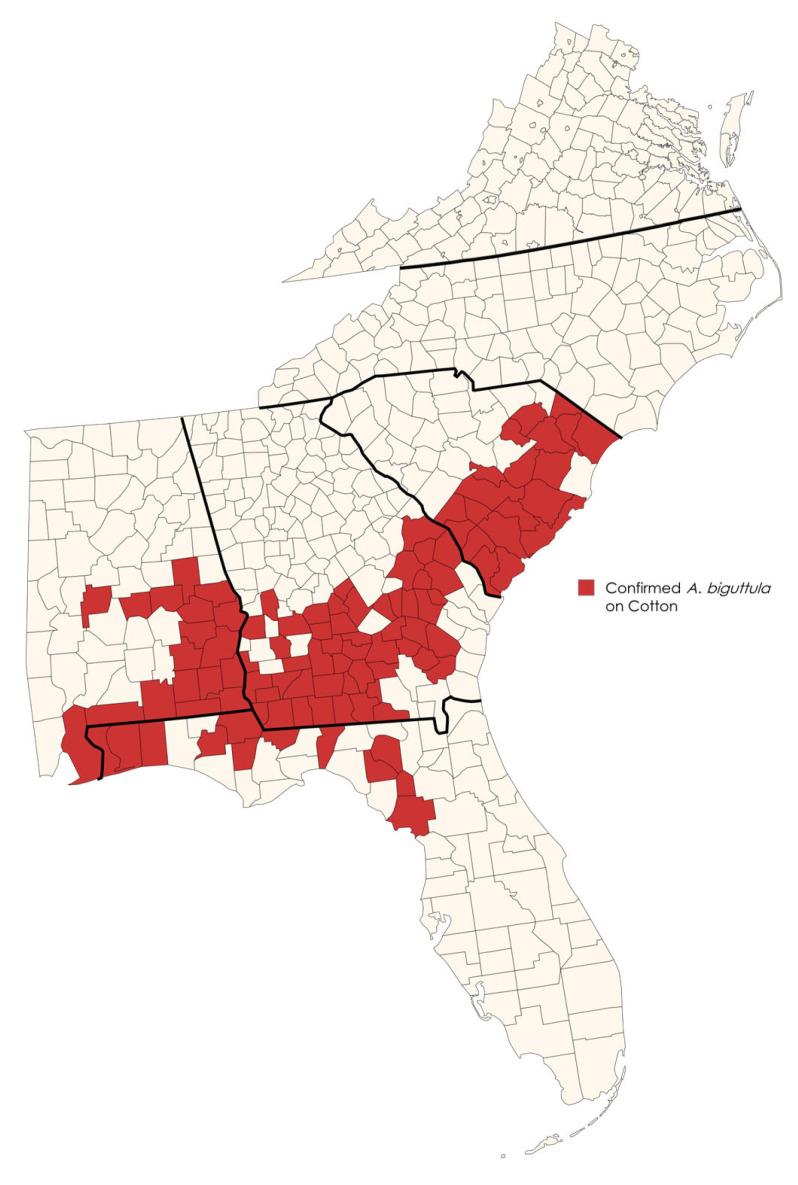
Counties in red indicate the detection of *A. biguttula* on cotton in Alabama, Florida, Georgia, South Carolina, North Carolina, and Virginia in the southeastern United States. Map created by I.L. Esquivel using https://www.mapchart.net/ (accessed on 25 Aug 2025).

**Figure 3:**
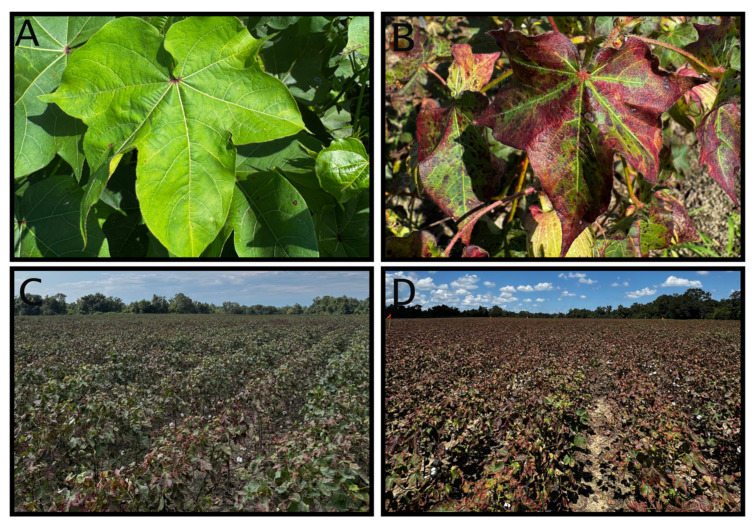
Various degrees of hopperburn symptoms seen in cotton. Slight yellowing on a single cotton leaf (**A**), late-stage reddening on a cotton leaf (**B**), severe case of hopperburn with green foliage present on a commercial field (**C**), dead leaves and initial defoliation four days after visit to the same commercial field (**D**).

Within four weeks, a coordinated survey effort detected *A. biguttula* in 101 counties across Alabama (18), Florida (12), Georgia (51), and South Carolina (20) on commercial and experimental cotton as of 29 August 2025 (Figure 2). The distance from the initial location of detection on cotton by Gilchrist Co., Florida, to the most northern infested field is approximately 645 km. Virginia and North Carolina were the only states in southeast America where *A. biguttula* were not detected during this initial cotton sampling. Cotton injury, hopperburn, was also detected in all locations at variable degrees of severity (Figure 3).

## 4. Discussion

*Amrasca biguttula* belongs to the tribe Empoascini, the second largest within the microleafhopper subfamily Typhlocybinae (Hemiptera: Cicadellidae), which contains approximately 1372 species worldwide [13,14]. In North America, most Empoascini are translucent green, similar to *A. biguttula*, and may be mistaken for similar pest species such as the potato leafhopper, which is a pest of peanut in the region. However, this species can be distinguished from most native congeners by its diagnostic external markings, two black dots on the apex of each forewing and a pair of small black spots on the crown of the head (Figure 1A). Definitive identification requires examination of male abdominal and genital characteristics [13,15]. To further complicate identification, *A. biguttula* is often referred to as *Amrasca devastans*, *Epoasca devastans*, and 14 other names in the scientific literature; however, a recent revision of the genus has clarified *A. biguttula* as the proper description [15].

Following initial detection, relatively low populations were found in surveyed fields, mainly composed of adults. However, in Gilchrist County, FL, both nymphs and adults were present, suggesting colonization for at least 2 weeks in the Florida panhandle (I.L.E, pers. obs.). The development time of A. biguttula varies from 8 to 46 days depending on the host plant, temperature, and relative humidity [16,17]. In cotton, the average nymphal development time (five instars) of *A. biguttula* from the first instar to adulthood is 8–11 days, completing a generation in roughly two weeks once adults colonize a field [18]. This could explain why initial populations appeared to be low, but visible injury was seen quickly after initial detection of adults. Given its small size and resemblance to similar native species, it is plausible that it was present in low numbers in Florida cotton and potentially neighboring states before detection and before populations were large enough for plant injury to be seen.

The potential impact of *A. biguttula* on 2025 cotton yield, or its extent of spread within the U.S. Cotton Belt, remains uncertain due to the late timing of detection. However, the rapid progression from detection to visible damage is concerning. Within less than two weeks, infested plants can deteriorate from slight discoloration to pronounced hopperburn symptoms and, in severe cases, defoliation (Figure 3). In India, *A. biguttula* populations in cotton typically peak between September and October, aligning with much of the cotton growing season in the southeastern United States [19]. Further, *A. biguttula* is active year-round through winter, feeding on okra, other winter vegetable crops, and at least 24 other reproductive host plants [20].

If *A. biguttula* exhibits similar phenology and population dynamics in the southeastern U.S., it could emerge as a recurring and economically significant pest of cotton, multiple vegetable crops, and ornamental plants. A recent climate modeling study showed that the southeastern United States was a suitable region for establishment, with increasing suitability under future climate models [21]. Further, there is potential for the dispersal of *A. biguttula* outside of the southeastern U.S. Some leafhopper species have been well documented to travel long distances via synoptic weather patterns [22,23]. For example, the potato leafhopper overwinters in the southern U.S states along the Gulf of Mexico and migrates to northern and eastern U.S. with the occurrence of warm, long-distance southerly winds [24].

## 5. Conclusions

The detection of *A. biguttula* across the southeastern cotton-growing region represents the first confirmed occurrence of this pest on cotton in the continental United States. While its current distribution appears limited, the pest’s rapid feeding injury, broad host range, dispersal potential, and potential for multiple generations per year raise concern over its spread and impact across the U.S. Cotton Belt. Even modest yield losses could translate to substantial economic consequences in an already stressed cotton industry. Coordinated monitoring and early detection will be critical to mitigate potential damage to cotton and other susceptible crops in the region.

In terms of management, economic thresholds developed in Pakistan and India for *A. biguttula* in cotton are variable and range from 1 to 5 per leaf [25]. Insecticide resistance in *A. biguttula* has been reported for multiple classes of commonly used products in the United States cotton industry, including pyrethroids (cypermethrin, bifenthrin), organophosphates (acephate), and neonicotinoids (imidacloprid, thiamethoxam, acetamiprid) [26,27,28]. However, labeled rates and concentrations may be different outside the U.S, and previous exposure will also influence *A. biguttula* management in the U.S. In addition to research on accurate economic thresholds and effective chemical control options, information on whether this pest will overwinter in the United States, and, if so, what potential hosts may allow them to survive and serve as a bridge between not only cotton but also other crop hosts is required.

## Data Availability

Specimens and data supporting the conclusions of this article will be made available by the authors on request.
